# Exploring the Psychological Mechanism of How the Multidimensional Service Quality of Fitness Centers Affects Consumer Satisfaction and Loyalty Depending on the Level of Exercise Involvement

**DOI:** 10.3390/bs14111049

**Published:** 2024-11-06

**Authors:** Manmin Kim, Daehwan Kim

**Affiliations:** 1 Department of Sports Rehabilitation and Training, Kyungnam College of Information & Technology, Busan 47011, Republic of Korea; ifpabusan@gmail.com; 2 Division of Smart Healthcare, Pukyong National University, Busan 48513, Republic of Korea

**Keywords:** fitness consumer, core service, physical servicescape, social servicescape, price fairness, flow experience, rapport formation, satisfaction, loyalty

## Abstract

This study aimed to examine the influence of core services and the physical servicescape and social servicescape on consumer satisfaction and loyalty via perceived price fairness, flow experience, and rapport in fitness centers. Additionally, the present study explored how exercise involvement moderates the relationship between perceived price fairness, flow experience, rapport, and consumer satisfaction. For these purposes, targeting adults using fitness centers in South Korea, a total of 763 participants were sampled based on a cross-sectional design and used for data analyses involving confirmatory factor analysis for measurement validation and latent moderated structural equation modeling for hypothesis testing. The results showed that the core services, physical servicescape, and social servicescape enhance perceived price fairness, flow experiences, and rapport between staff and consumers, respectively. The study findings also indicated that exercise involvement significantly moderates the effect of price fairness and flow experience on satisfaction but not rapport. These insights offer practical and theoretical implications for fitness center management, emphasizing the importance of tailored service strategies in retaining loyal customers.

## 1. Introduction

As the importance of physical activity and the pursuit of a healthy lifestyle have become prominent, the global fitness industry has grown significantly [1]. According to Fortune Business Insights [2], the fitness center market size was estimated to be USD 104.05 billion in 2022, and it is expected to grow at an average annual rate of 8.83%, reaching a market size of USD 202.07 billion by 2030. Amid this remarkable growth, fitness center managers find themselves in fierce competition to attract new customers and retain existing ones. The intense competition in the fitness industry is particularly pronounced in South Korea. For instance, despite over 10% average annual growth in the Korean fitness industry over the past two decades, it has one of the highest closure rates among the arts, sports, and leisure service sectors, with an 81.6% business closure rate within five years of establishment [3]. Accordingly, providing useful insights into efficient strategies for the sustainable operation of fitness centers not only in South Korea but also in the world is imperative. In particular, to ensure the profitability and sustainability of fitness center businesses in such a competitive environment, it is crucial to focus on strategies prioritizing retaining existing customers (i.e., customer loyalty) over acquiring new ones.

In line with the rationale behind developing efficient strategies for ensuring the profitability and sustainability of fitness center businesses, scholars, using diverse perspectives, have investigated how customer loyalty can be ensured [4,5,6,7,8,9]. For example, drawing on the SERVQUAL model [10], Huang and Kim (2023) revealed that the service quality of fitness centers enhances consumer loyalty through satisfaction, trust, and commitment [6]. Although the existing literature offers meaningful insights into effective strategies for developing customer loyalty in the context of fitness centers, further investigation is needed to understand and explain how and when consumer loyalty can be effectively developed from a comprehensive perspective.

Generally, one of the most powerful determinants of customer loyalty is customer satisfaction [4,11]. Which factors enhance customer satisfaction, and how do they do so in the context of fitness centers? According to Chelladurai et al. (1987), fitness services mainly consist of core and peripheral services [12]. The existing literature shows that these service dimensions are major determinants of consumer satisfaction in the context of fitness centers, since they not only enhance the value perception of the services (e.g., price fairness) but also facilitate positive consumption experiences (e.g., flow experiences) [6,13,14,15,16]. Furthermore, previous research highlights the pivotal role of frontline employees (e.g., fitness trainers) in the success of service businesses in that they can form positive relationships with their customers (e.g., rapport), and such positive relationships contribute to consumer satisfaction [11,17]. Taken together, consumer satisfaction, which is a powerful determinant of consumer loyalty, is dynamically affected by diverse factors. Thus, it is necessary to systematically investigate the psychological mechanism of how such factors influence consumer satisfaction in the context of fitness centers.

Fitness consumers have a wide range of personal characteristics, and their decision-making processes tend to depend on such characteristics [6,13,18]. This implies that the psychological mechanism underlying consumer satisfaction is likely to show different patterns depending on consumers’ characteristics. Nevertheless, the majority of previous studies on fitness consumers’ satisfaction have overlooked the significant role of consumers’ characteristics in consumers’ decision-making processes. To fill the theoretical gap, exercise involvement as a personal characteristic should be considered when exploring the psychological mechanism of consumer satisfaction in the context of fitness centers because exercise involvement is a basis for fitness market segmentation [6].

Based on the aforementioned research rationales, the current study aimed to investigate the effects of the core service, servicescape, and employee quality on consumer satisfaction and loyalty via price fairness, flow experience, and rapport. Furthermore, this investigation sought to examine the moderating role of exercise involvement in the effects of price fairness, flow experience, and rapport on consumer satisfaction. The findings of this study are expected to expand the body of knowledge on consumer behaviors in the context of fitness centers and to provide useful insights into developing efficient strategies for the profitable and sustainable operation of fitness centers.

## 2. Theoretical Background

### 2.1. Service Dimensions in Fitness Centers

The concept of service quality [10] has been widely used to understand and explain consumer behaviors in the context of sports and fitness centers [6,14,19,20,21,22]. For example, Huang and Kim (2023) conceptualized the service quality of sports centers as the perceived reliability, tangibility, assurance, empathy, and responsiveness of services offered by these centers and found that these service components significantly influence consumer loyalty via satisfaction, trust, and commitment [6]. Although the concept of service quality offers useful insight into the importance of managing the quality of services provided by fitness centers, it is unclear which service dimensions (e.g., programs, physical environments, and employees) are particularly important for enhancing consumer satisfaction and loyalty.

Chelladurai [12] argued that fitness centers commonly offer diverse services which are not necessarily complementary to each other, and he suggested that their services are dichotomized as primary and secondary dimensions. Specifically, primary dimensions refer to all aspects of the services related to fitness per se, while secondary dimensions refer to those goods or services which do not relate to fitness. In line with this notion, the existing literature has suggested two relevant concepts: core and peripheral services [23,24,25,26,27]. Generally, core services represent the essential features of a service, such as fitness testing and instruction, whereas peripheral services denote any factors surrounding and completing the core services [12,23].

While the existing literature suggests a consistent conceptualization of core services in the context of fitness centers, the conceptualization of peripheral services is relatively unclear [13,28,29,30,31,32]. The majority of previous studies have used the concept of a servicescape [33] as peripheral services to explain fitness consumer behaviors, and they have mainly focused on the physical aspects of the servicescape. However, researchers have argued that it is necessary to expand the concept of the servicescape from a focus on the physical environment to social domains [34,35,36,37]. For example, Tombs and McColl-Kennedy (2003) pointed out the limitations of traditional servicescapes, noting that the relationship quality between customers and employees can influence customers’ cognitive and emotional experiences in diverse ways, thus suggesting a conceptual model of a social servicescape [35]. Furthermore, Wang and Chiu (2022) examined the effect of interaction between fitness customers and employees on service quality and highlighted the role of the service encounter in consumer loyalty in the context of fitness centers [38]. Based on the discussion above, the present study conceptualized the peripheral services of fitness centers with the physical environment (i.e., servicescape) and social domains (i.e., social servicescape).

Specifically, the concept of the servicescape refers to any tangible factors of a service provider which affect consumer perceptions or behaviors, and its conceptualization depends on the target service context [13,29]. In the context of fitness centers, the conceptualization of a servicescape mostly involves spatial layout, aesthetics, convenience, ambient conditions, and cleanliness [13,28,39,40].

The term spatial layout describes the organization of furniture and equipment within a service area, focusing on their spatial relationships.

Aesthetics pertain to elements such as interior and exterior design, decor, and architectural style, all of which enhance the visual appeal of a service facility. Further, ambient conditions include background elements of a service environment, such as air quality, temperature, lighting, and music, which influence consumers’ sensory experiences. Lastly, cleanliness refers to maintaining a clean and sanitary environment within a fitness center.

Furthermore, the concept of a social servicescape in the context of fitness centers refers to the social environment within a service setting, including interactions between customers and staff as well as the social atmosphere created by these interactions [41]. This concept expands upon the traditional notion of the servicescape, which focuses primarily on the physical environment (e.g., layout, aesthetics, and ambient conditions) of service settings [33]. In fitness centers, the social servicescape includes elements such as the physical appearance, friendliness, and professionalism of staff [35]. The environment can significantly impact members’ satisfaction, loyalty, and overall motivation to continue using a fitness facility [42]. In summary, the present study conceptualizes service dimensions as core and peripheral services, which include physical and social servicescapes.

### 2.2. Effects of Core Service, Physical Servicescape, and Social Servicescape

The multi-dimensional services provided by fitness centers play a pivotal role in consumer perceptions, experiences, evaluations, and decision making. First, the concept of perceived price fairness refers to consumers’ evaluation of whether the price of a service is justified based on the value received [43]. In the context of fitness centers, core services such as exercise programs, personal training, and fitness tests play a crucial role in shaping customers’ perceptions of the value they receive for the money spent. According to Zeithaml (1988), perceived value is defined as a consumer’s overall assessment of the utility of a product based on perceptions of what is received and what is given [44]. Fitness centers offering high-quality core services can enhance perceived value, thereby positively influencing customers’ perceptions of price fairness. In other words, when customers perceive that they are receiving high-quality services, they are likely to view the prices charged by the fitness center as fair [45]. Moreover, the concept of service quality, particularly in the context of fitness centers, is often evaluated based on the effectiveness and variety of the core services provided [46]. High-quality exercise programs tailored to the needs and preferences of members can significantly enhance a perception of fair pricing, leading to customer satisfaction and loyalty [47]. Therefore, the positive impact of core services on perceived price fairness can be explained by the customers’ perceived value derived from high-quality and effective fitness programs, and such a price perception would lead to customer satisfaction.

Next, in the context of fitness centers, the physical servicescape, including spatial layout, aesthetics, convenience, ambient conditions, and cleanliness, significantly affects consumers’ perceptions, emotions, and ultimately their level of engagement in exercise activities (i.e., flow experience) [42]. Exercise flow is a psychological state of complete absorption, time distortion, and enjoyment during physical activity [48]. Flow theory posits that certain environmental conditions can facilitate or hinder an individual’s ability to achieve this state of immersion and engagement [49]. The relationship between the physical servicescape and exercise flow can be explained by the environmental psychology framework, which suggests that the physical environment influences emotional responses and behavior [50]. A well-designed servicescape in fitness centers can enhance consumers’ sensory experiences, reduce perceived effort, and create a conducive environment for achieving an exercise flow state. For instance, factors such as adequate lighting, a comfortable temperature, and appropriate music can create a motivating atmosphere which encourages consumers to engage deeply in their workouts, leading to a state of flow. A positive servicescape, including cleanliness, spaciousness, and modern equipment, contributes to high satisfaction and motivation, which are essential components of flow in exercise contexts [13]. In summary, a well-maintained and aesthetically pleasing physical environment in fitness centers can significantly enhance exercise experiences by facilitating the flow state, ultimately leading to great exercise commitment and satisfaction.

Lastly, the social environment can significantly impact the overall service experience and satisfaction. In fitness centers, the social servicescape involves interactions between the fitness staff (e.g., trainers) and consumers. These interactions are critical in forming a rapport, which is defined as a harmonious relationship characterized by mutual understanding and trust [17]. According to the social interaction theory, the quality of interpersonal relationships in service settings plays a significant role in shaping customer perceptions and attitudes [35]. In fitness centers, effective communication, friendly behavior, and personal attention from staff can foster a sense of belonging and trust, which are essential elements of a rapport [51]. Moreover, such social connections contribute to customers’ overall satisfaction and willingness to maintain long-term memberships [17,51]. Additionally, the service-dominant logic framework [52] highlights the co-creation of value in service environments, emphasizing the importance of social interactions in shaping customer experiences and loyalty. Based on the discussion thus far, the following hypotheses were developed:

**H1.**  *Core services positively influence perceived price fairness.*

**H2.**  *The physical servicescape positively influences exercise flow experience.*

**H3.**  *The social servicescape positively influences rapport formation between consumers and staff.*

Furthermore, perceived fairness of pricing significantly affects customer satisfaction and loyalty in the context of fitness centers [43]. When customers perceive a service price as being fair, they are likely to feel positive about the value they receive from the service, which enhances overall satisfaction [53]. Fair pricing in fitness centers, such as reasonable membership fees, transparent payment plans, and value-added services, creates a sense of trust, reducing any perceived inequity in a transaction and increasing customer satisfaction [54]. Moreover, pricing fairness is particularly critical for service industries such as fitness centers, where the intangibility of the service can lead to uncertainties about value [44]. Thus, when consumers believe that a fitness center charges fairly, their perception that they are receiving good value for their money is reinforced, resulting in high satisfaction [55].

In fitness centers, achieving an exercise flow state, where individuals are fully absorbed in their workout, contributes to positive emotional experiences and enhances overall satisfaction with the fitness center [13,56]. Research has shown that a physical servicescape, such as well-maintained equipment, comfortable layout, and ambient conditions, can significantly contribute to the creation of exercise flow by reducing distractions and enhancing the workout environment [33]. When customers experience flow during their fitness routines, they are likely to associate positive emotions with the fitness center, resulting in increased satisfaction [57,58].

Rapport formation between fitness center staff and consumers is another key factor influencing satisfaction. In service environments, especially those requiring regular and close interactions such as fitness centers, the rapport between staff and customers can significantly enhance the overall service experience [51]. The social servicescape model [35] suggests that the social dimension of a servicescape, such as interactions between customers and staff, plays a crucial role in shaping customer satisfaction. Positive interactions which foster rapport create a supportive and friendly atmosphere, making customers feel valued and cared for, which in turn leads to high levels of satisfaction [59]. The following hypotheses were developed:

**H4.**  *Perceived price fairness positively influences consumer satisfaction.*

**H5.**  *Exercise flow experience positively influences consumer satisfaction.*

**H6.**  *Rapport formation between consumers and staff positively influences consumer satisfaction*.

### 2.3. Exercise Involvement as a Consumer Characteristic

Exercise involvement refers to the extent to which individuals integrate exercise into their lives as a central and meaningful activity. According to the involvement theory, individuals who are deeply involved in an activity are likely to exhibit consistent and loyal behavior toward it [60]. For example, individuals who perceive exercise as a central component of their lifestyle attach a high value to their engagement with fitness-related activities, which in turn shapes their satisfaction and loyalty. Consumers with high exercise involvement are likely to critically evaluate their fitness center experience and place great importance on the attributes of the fitness center which contribute to their engagement. In this respect, exercise involvement can moderate the relationship between perceived price fairness and consumer satisfaction. Research suggests that consumers with high involvement levels tend to be satisfied when they perceive the price of services to be fair because they are attuned to the quality and benefits derived from fitness centers [60,61].

Furthermore, exercise involvement moderates the relationship between exercise flow and consumer satisfaction in fitness centers. For individuals with high exercise involvement, flow experiences are critical to their overall satisfaction with a fitness center. This is because highly involved individuals prioritize their exercise experiences and are likely to view the flow state as an essential factor in determining whether a fitness center meets their expectations. When flow is achieved, they associate these positive experiences with the environment, increasing their satisfaction [60,62]. Conversely, individuals with low exercise involvement may not prioritize the flow state as much, and their satisfaction may significantly depend on external factors such as convenience or accessibility. The degree to which flow influences satisfaction can vary significantly depending on an individual’s level of exercise involvement, making involvement a key moderating variable in the relationship between flow and satisfaction [63].

Lastly, highly involved consumers tend to have a strong connection with fitness activities, making them receptive to social interactions with staff in aspects such as personalized communication and rapport-building efforts. Consumers with high exercise involvement are likely to value rapport because their engagement with fitness activities makes them expect a holistic service experience. For these consumers, a rapport with staff can lead to great overall satisfaction with a fitness center because they regard the relationship with the staff as integral to their fitness journey. Conversely, consumers with low exercise involvement may prioritize functional aspects of the fitness center (such as equipment and cleanliness) over rapport, reducing the influence of rapport on satisfaction for them [61]. All in all, the following hypotheses were developed:

**H7.**  *Exercise involvement positively moderates the effect of perceived price fairness on consumer satisfaction.*

**H8.**  *Exercise involvement positively moderates the effect of exercise flow experience on consumer satisfaction.*

**H9.**  *Exercise involvement positively moderates the effect of rapport formation on consumer satisfaction.*

### 2.4. Consumer Satisfaction and Loyalty

The relationship between consumer satisfaction and loyalty is well established in service industries, including fitness centers. In this context, consumer satisfaction refers to the overall positive evaluation of services provided, while loyalty encompasses the consumer’s intention to return and recommend a service to others. Satisfied consumers tend to exhibit great loyalty, often demonstrated through membership renewals and positive word-of-mouth recommendations. According to Oliver (1999), satisfaction is a precursor to loyalty, particularly when it evolves into a long-term commitment [64]. In fitness centers, MacIntosh and Doherty (2007) emphasized that satisfied consumers are likely to continue their memberships, engage with a brand, and promote it within their social circles [24]. Furthermore, Alexandris et al. (2004) found that satisfaction with fitness services, including both physical and social components, significantly predicts consumer loyalty in the health and fitness industry [65]. Accordingly, the following hypothesis was formulated:

**H10.**  *Consumer satisfaction positively influences consumer loyalty.*

**Figure 1:**
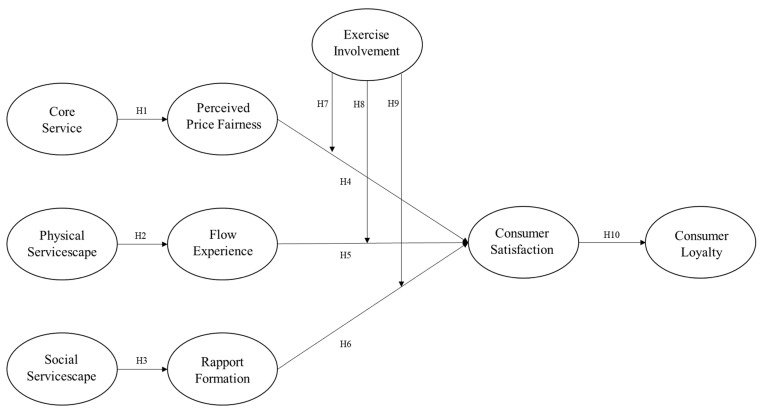
Research model.

A research model was developed based on the hypotheses (Figure 1).

## 3. Methods

### 3.1. Participants

To achieve the research purpose and test the theoretical relationship between constructs in the research model, we conducted an empirical study based on post-positivism. This study targeted adults using fitness centers in South Korea. The study employed a cross-sectional research design to collect data. Specifically, a professional online survey institution (https://embrain.com/), which holds 1,710,000 panels across the country, was commissioned to randomly select participants. The survey link was distributed to 800 participants, and after excluding 37 invalid or incomplete responses, data from 763 participants were used for the final analysis. Descriptive statistics revealed that 380 participants (49.8%) were male, with an average age of 42.1 years (SD = 13.31). Detailed demographic information is presented in Table 1.

### 3.2. Instruments

We measured 9 constructs using existing measurement items on 7 point Likert scales (1 = “not at all”; 7 = “very much”). Specifically, we measured the construct of core services using four measurement items adapted from Chelladurai (1987) [12]. The measurement items for the physical servicescape, including the spatial layout (5 items), aesthetics (4 items), convenience (4 items), ambient conditions (5 items), and cleanliness (5 items), were adapted from the existing literature on servicescapes [13,28,30,33,39]. We measured the concept of the social servicescape, including the appearance (4 items), friendliness (4 items), and professionalism (4 items) of staff, with measurement items adapted from the existing literature on social servicescapes [35,47].

To measure perceived price fairness, we adapted five items from previous studies on price fairness [66,67] and modified the items to fit the research context. We measured exercise flow experience using six items adapted from the existing literature on flow experience [18,49,56,68,69]. Furthermore, rapport formation was measured using five items adapted from Gremler and Gwinner (2000) [17].

Exercise involvement was measured using three items adapted from Zaichkowsky’s research [51]. Furthermore, we measured consumer satisfaction using three items and consumer loyalty using five items from the existing literature on consumer satisfaction and loyalty in the context of fitness centers [6,13]. Taken together, 66 items were included in the specified measurement model. Lastly, the survey items were translated into Korean by two bilingual authors and then back translated into English. The authors compared the original version with the translated version and resolved minor differences through discussion and agreement to develop the final measurement items.

### 3.3. Data Analysis

Before the hypothesis testing, we performed a descriptive statistical analysis to explore research participants’ demographic information and find potential outliers. Then, we conducted a confirmatory factor analysis (CFA) to investigate the reliability and validity of the specified measurement model using Mplus 8. Finally, to test the research hypotheses, a latent moderated structural (LMS) equation analysis was performed based on the two-step approach suggested by Klein and Moosbrugger [70]. Specifically, a structural model without any interaction terms was estimated and assessed based on traditional fit indices in the first step. Once the structural model met the fit indices, another model with latent interaction terms was estimated and evaluated based on a log-likelihood difference test between the two models in the second step. If a statistically significant difference between the two models was found, then the path coefficients in the model with the latent interaction terms were used to test the research hypotheses.

## 4. Results

### 4.1. Measurement Model Validation

We performed a CFA to evaluate the reliability and validity of the theorized measurement model. The results of the CFA indicated an acceptable fit between the measurement model and data (χ^2^/df = 3166.930/1608 = 1.969; CFI = 0.946; TLI = 0.943; RMSEA = 0.036; SRMR = 0.040). All standardized factor loadings of the measurement model were found to be statistically significant and greater than 0.50 (Table 2). The calculated values of the average variance extracted (AVE) ranged from 0.60 (core service) to 0.79 (perceived price fairness). Furthermore, the composite reliability coefficients of each latent factor were greater than 0.80. These results ensured the reliability and convergent validity of the measurement model. Lastly, the AVE values were found to be greater than the squared correlation coefficients between all latent factors except for the correlations between the core service, social servicescape, and consumer satisfaction, ensuring the marginal discriminant validity of the measurement model (Table 3). The high correlations between the core service, social servicescape, and consumer satisfaction may indicate discriminant validity issues in the measurement model. Hence, we performed a more sensitive evaluation of discriminant validity using heterotrait-monotrait ratio of correlations (HTMT) [71], which is an index of similarity between constructs. Specficially, if an HTMT value between two constructs is smaller than one, then the pair of these constructs has discriminant validity [71]. The results of the HTMT test showed that all HTMT values were below the threshold of one. All in all, it can be concluded that the specified measurement model secures reliability and validity [72,73].

### 4.2. Hypothesis Testing

To test the research hypotheses, LMS analysis was conducted based on the two-step approach suggested by Klein and Moosbrugger [70]. Specifically, we estimated a structural model without the latent interaction factors (perceived price fairness × exercise involvement, exercise flow × exercise involvement, and rapport formation × exercise involvement) in the first step. The results indicate that the structural model fit the data (χ^2^/df = 3602.246/1630 = 2.210; CFI = 0.932; TLI = 0.929; RMSEA = 0.040; SRMR = 0.063). Hence, we proceeded to the second step and estimated another structural model with the latent interaction factors (perceived price fairness × exercise involvement, exercise flow × exercise involvement, and rapport formation × exercise involvement). Additionally, we conducted a log-likelihood difference test between the estimated models (log-likelihood ratio test: Klein and Moosbrugger [70] and Maslowsky et al. [74]). The results show that the structural model with the latent interaction factors was statistically superior to the one without them (D = 2[|−54482.480| − |−54503.630|] = 42.30; Δdf = 3). Accordingly, the present study utilized the path coefficients in the structural model, including the latent interaction factors, to test the research hypotheses.

The results indicated that the core service has a positive impact on perceived price fairness (γ = 0.669; *p* < 0.001). Furthermore, the physical servicescape was found to have a positive effect on exercise flow experience (γ = 0.706; *p* < 0.001). Additionally, the social servicescape was found to positively influence rapport formation (γ = 0.682; *p* < 0.001). All in all, H1, H2, and H3 were supported.

Next, the results show that perceived price fairness (β = 0.642; *p* < 0.001), exercise flow experience (β = 0.353; *p* < 0.001), and rapport formation (β = 0.150; *p* < 0.001) had positive effects on consumer satisfaction. Hence, H4, H5, and H6 were tenable. Regarding the moderating effect of exercise involvement, the latent interaction factors between perceived price fairness and exercise involvement (γ = 0.153; *p* < 0.001) and the one between exercise flow experience and exercise involvement (γ = −0.117; *p* < 0.01) significantly influenced consumer satisfaction. However, the latent interaction factor between rapport formation and exercise involvement did not significantly influence consumer satisfaction (γ = 0.061; *p* = 0.076). Notably, despite the significant effect of the latent interaction factor between flow experience and exercise involvement on consumer satisfaction, its direction (negative) was found to be different from the hypothesis (positive). Accordingly, H7 was supported, while H8 and H9 were rejected. Lastly, consumer satisfaction positively affected consumer loyalty (β = −0.851; *p* < 0.001), and thus H10 was accepted. The results of the hypothesis testing are summarized in Table 4.

## 5. Discussion

### 5.1. Interpretation of Results

The findings of the present study offer a comprehensive view of the factors contributing to consumer satisfaction and loyalty within the context of fitness centers. Specifically, one of the key findings of this study is that perceived price fairness has a positive effect on core services. This result supports previous research, which suggests that when consumers perceive that they are receiving high-quality core services, they are likely to regard the prices of those services as fair [44,45]. In fitness centers, core services such as personal training, group exercise programs, and fitness testing are central to consumer experience. The quality of these core services forms the foundation upon which customers evaluate whether the price they are paying is justified. This result aligns with Howat et al.’s (1996) findings, reporting that service quality is a critical determinant of consumer perceptions of value [46]. Fitness centers which offer personalized and high-quality core services can enhance perceived value, which in turn influences the consumer’s perception of price fairness. This result also confirms the findings of previous research conducted in the context of health and fitness services, where the effectiveness of core services was found to be a key driver of customer satisfaction and loyalty [12,29].

This study also revealed that the physical servicescape of fitness centers has a significant positive effect on the exercise flow experience. This finding is consistent with the environmental psychology framework, which posits that the physical environment can significantly influence emotional responses and behavior [50]. In this context, physical aspects such as the spatial layout, cleanliness, and ambiance play a crucial role in creating an environment conducive to achieving flow during exercise. Flow theory [48] suggests that flow experiences occur when individuals are fully absorbed in an activity and experience a state of effortless concentration. For fitness centers, achieving flow is critical because it not only enhances consumers’ workout experience but also increases their overall satisfaction with a facility. Previous research by Jeon et al. (2021) found that factors such as spaciousness, cleanliness, and aesthetic appeal contribute to the creation of a flow experience by minimizing distractions and promoting engagement in an activity [13].

The present study found that the social servicescape, including the quality of interactions between staff and consumers, significantly contributes to rapport formation. Rapport is an essential component of the service experience in fitness centers. When consumers feel that staff are friendly, professional, and attentive to their needs, they are likely to develop rapports, which leads to great satisfaction. The findings of the present study support the social interaction theory, which posits that the quality of interpersonal relationships in service settings plays a pivotal role in shaping customer perceptions and attitudes [35]. In fitness centers, rapport formation is particularly important because the interaction between staff and consumers is frequent and personalized. A demonstration of expertise, provision of encouragement, and fostering of positive social interactions by staff can significantly enhance consumers’ overall satisfaction with a fitness center. Previous research has highlighted the importance of staff interactions in shaping customer experiences. For example, Price and Arnould (1999) found that rapport significantly contributes to customer satisfaction by creating a supportive and friendly atmosphere [51].

The current study found that exercise involvement positively moderates the relationship between perceived price fairness and consumer satisfaction. For highly involved consumers, price fairness has a strong positive effect on satisfaction. This finding is consistent with previous research by Beaton et al. (2011) and Iwasaki and Havitz (2004), which suggested that highly involved individuals are likely to derive satisfaction from services which align with their values and interests [60,61]. In this case, fitness centers which provide high-quality services at a fair price are likely to satisfy highly involved consumers, who are attuned to the quality of the services they receive.

This study found a negative moderating effect of exercise involvement on the relationship between exercise flow and consumer satisfaction. This result contradicts the initial hypothesis, which expected a positive moderating effect. A possible explanation for this unexpected finding could be that for highly involved individuals, the flow state might be expected or routine, reducing the impact of the flow state on their overall satisfaction with the fitness center. Consequently, flow experiences may not be as strongly linked to their satisfaction, as this is for individuals with low involvement, who may find flow novel and engaging.

Lastly, exercise involvement does not significantly moderate the relationship between rapport formation and consumer satisfaction. While rapport formation remains important for overall satisfaction, this finding suggests that the level of exercise involvement may not significantly affect the degree to which consumers value rapports with fitness center staff. This outcome could be due to the fact that social interactions with staff are generally appreciated by all consumers, regardless of their level of involvement in fitness activities.

As expected, this study confirmed the strong positive relationship between consumer satisfaction and consumer loyalty. Satisfied consumers are likely to remain loyal to a fitness center, renew their memberships, and recommend the center to others. This finding is consistent with previous research, which has shown that satisfaction is a key determinant of loyalty in service industries [64,65]. In the context of fitness centers, satisfaction is driven by a combination of factors, including the quality of core services, the physical environment, and the quality of social interactions with staff. Consumers who are satisfied with these aspects of a fitness center are likely to continue using it and recommend it to others.

### 5.2. Theoretical and Practical Implications

This study makes several theoretical contributions to the literature on consumer behavior in fitness centers. First, by integrating the core services, physical servicescape, and social servicescape into a single framework, this investigation broadens the present understanding of how these multidimensional aspects of service quality influence consumer satisfaction and loyalty. Traditionally, studies have examined these aspects separately, but this research emphasizes the interconnectedness of these aspects, offering a comprehensive view of the fitness center experience.

Second, this study highlights the critical role of exercise involvement as a moderating factor, offering new insights into how consumer characteristics influence service evaluations. Exercise involvement, typically explored in the context of sports and recreational activities, is shown here to impact the relationship between service dimensions and satisfaction, offering a rich understanding of how involvement shapes consumer behavior in a fitness setting. This moderating role of exercise involvement highlights the importance of considering personal characteristics when developing strategies to enhance consumer satisfaction.

Moreover, this research contributes to servicescape theory by expanding its conceptualization beyond physical factors to include social dimensions (i.e., social servicescape). While prior studies have mainly focused on the physical environment of service settings, this study aligns with emerging research advocating for a holistic approach to servicescapes, incorporating social interactions between staff and consumers as a vital element. This approach provides a deep understanding of how rapport building between staff and customers impacts overall satisfaction with services.

This study also offers new insights into price fairness theory in the context of fitness centers. Furthermore, this research reinforces the idea that perceived price fairness directly impacts consumer satisfaction, which is particularly relevant in service industries where the intangibility of a product often creates uncertainty about value. This research adds to the existing literature by demonstrating how core services, such as exercise programs and personal training, shape consumers’ perceptions of fairness, ultimately influencing their satisfaction and loyalty.

Lastly, this study contributes to the growing body of work on flow theory by demonstrating the importance of flow experience in exercise settings. The finding that exercise flow contributes significantly to consumer satisfaction supports the idea that creating environments conducive to flow is essential for service providers. This finding has implications for enhancing consumer engagement and satisfaction, especially in fitness centers, where the quality of the physical environment plays a crucial role in facilitating such experiences.

The findings of this study provide several actionable insights for fitness center managers aiming to enhance customer satisfaction and loyalty. First, managers should prioritize core services such as exercise programs and personal training, ensuring high quality and customization to meet customer expectations. Offering superior core services justifies pricing strategies and enhances perceived price fairness, leading to high satisfaction.

Second, the physical servicescape, including spatial layout, cleanliness, aesthetics, and ambient conditions, must be well maintained. A conducive environment for exercise flow encourages members to feel engaged in their workouts, which in turn fosters positive emotional responses and overall satisfaction. Fitness center managers should consider investing in well-designed, clean, and aesthetically pleasing facilities to support the exercise experiences of their customers.

Third, the social servicescape, especially the rapport between staff and members, plays a significant role in shaping satisfaction. Frontline employees such as trainers need to be personable, attentive, and skilled in fostering positive social interactions. Managers can invest in customer service training to help staff build strong relationships with members, thereby increasing the likelihood of long-term membership retention and word-of-mouth recommendations.

Finally, managers should tailor their strategies according to the level of exercise involvement among members. Highly involved members are likely to evaluate a fitness center critically, paying attention to both the quality of services and the fairness of pricing. For this segment, centers should offer services such as advanced personal training programs and detailed fitness assessments which match their high expectations. In contrast, consumers with low involvement may prioritize exercise experience formed by the physical servicescape, requiring different engagement strategies.

### 5.3. Limitations and Future Research Agenda

This study, while offering significant insights, has limitations. First, the research was conducted within a specific cultural and geographical context, namely South Korean fitness centers. Therefore, the generalizability of the findings to other regions or countries with different fitness cultures or consumer behaviors is limited. Future research should replicate this study in diverse cultural contexts to examine the external validity of the structural research model.

Second, this study focused primarily on three service dimensions—core service, physical servicescape, and social servicescape—as drivers of consumer satisfaction. While these are important factors, other dimensions such as technological integration (e.g., fitness apps and smart equipment), emotional responses, and psychological benefits from fitness center usage were not considered. Including these dimensions in future research could offer a holistic understanding of the elements which contribute to consumer satisfaction and loyalty in fitness centers.

Lastly, this study found that exercise involvement moderates the relationship between price fairness, flow experience, and rapport formation with consumer satisfaction. However, the moderating effect was not significant for rapport formation, indicating that the role of involvement in social dynamics within fitness centers requires further exploration. Future studies could investigate whether other consumer characteristics, such as personality traits, social orientation, or exercise goals (e.g., fitness, weight loss, and social interaction), influence the relationship between rapport and satisfaction. Moreover, the negative moderating effect of exercise involvement on the impact of flow experience on satisfaction suggests a complex relationship which warrants additional investigation. Understanding why highly involved consumers might not benefit as much as they would from flow in terms of satisfaction would be a valuable avenue for future research.

## 6. Conclusions

In conclusion, this study contributes valuable insights into the factors influencing consumer satisfaction and loyalty in the fitness center context. By examining the core services, physical servicescape, and social servicescape as key service dimensions, this research demonstrated their significant roles in shaping consumer perceptions of price fairness, exercise flow, and rapport formation. Additionally, the moderating effect of exercise involvement highlights the complexity of consumer behavior, showing that highly involved individuals evaluate fitness experiences differently than consumers with low involvement. Moreover, this research underscores the importance of tailored strategies in fostering consumer loyalty, emphasizing the need to provide high-quality services, maintain a conducive physical environment, and build strong consumer–staff relationships. However, limitations such as the focus on a single cultural context and the omission of other potentially relevant variables suggest that future research should expand on these findings. Broad cross-cultural studies and the inclusion of psychological and technological factors could further enrich the understanding of how fitness centers can enhance consumer satisfaction and loyalty in a competitive industry.

## Figures and Tables

**Table 1:** Demographic information.

Variable	Group	*n*	%
Gender	Male	380	49.8%
	Female	383	50.2%
Education Level	High school diploma or below	130	17.0%
	Associate’s degree	81	10.6%
	Bachelor’s degree	457	59.9%
	Master’s degree or above	95	12.5%
Occupation	Student	56	7.3%
	Office worker	390	51.1%
	Civil servant	38	5.0%
	Professional	89	11.7%
	Self-employed	57	7.5%
	Housewife	83	10.9%
	Etc.	50	6.6%
Exercise Participation per Week	Once a week	37	4.8%
	Twice a week	179	23.5%
	Three times a week	322	42.2%
	Four times a week	137	18.0%
	Five or more times a week	88	11.5%
Total		763	100%

**Table 2:** Summary of results for confirmatory factor analysis (CFA).

Factors and Items	λ	CR	AVE
Servicescape (Spatial Layout)		0.90	0.65
The size of the fitness center is appropriate	0.80		
The space between the fitness equipment is sufficient	0.84		
The fitness equipment is well arranged	0.80		
Moving around the fitness area is convenient	0.74		
There is enough space for exercising in the fitness center	0.86		
Servicescape (Aesthetics)		0.90	0.68
The exterior design of the fitness center is appropriate	0.74		
The interior design of the fitness center is appropriate	0.86		
The symbols and decorations in the fitness center are appropriate	0.84		
The design of the fitness equipment is appropriate	0.85		
Servicescape (Convenience)		0.86	0.61
The locker room in the fitness center is convenient to use	0.68		
The exercise area in the fitness center is convenient to use	0.78		
The amenities in the fitness center are convenient to use	0.84		
The fitness equipment is convenient to use	0.82		
Servicescape (Ambient Conditions)		0.89	0.62
The indoor color scheme of the fitness center is appropriate	0.80		
The indoor lighting of the fitness center is appropriate	0.81		
The indoor temperature of the fitness center is appropriate	0.80		
The indoor humidity of the fitness center is appropriate	0.82		
The indoor music in the fitness center is appropriate	0.70		
Servicescape (Cleanliness)		0.90	0.65
The fitness center is generally clean	0.83		
The restroom in the fitness center is clean	0.82		
The shower room in the fitness center is clean	0.80		
The fitness equipment is clean	0.85		
The supplies (uniforms and towels) at the sports center are clean	0.74		
Social Servicescape (Appearance)		0.86	0.67
The staff have shapely bodies	0.84		
The staff have an attractive appearance	0.76		
The staff wear appropriate clothing	0.85		
Social Servicescape (Friendliness)		0.91	0.78
The staff are always willing to help members	0.84		
The staff are genuinely interested in solving members’ problems	0.82		
The staff spend enough time addressing challenges	0.84		
Social Servicescape (Professionalism)		0.90	0.75
The staff display professional knowledge when answering my questions	0.87		
The staff skillfully demonstrate exercises	0.85		
The staff answer my questions clearly and simply	0.89		
Core Service		0.86	0.60
The fitness center provides superior exercise programs	0.78		
The fitness center provides fitness tests promptly	0.69		
The fitness center has an appropriate system in place to handle emergencies	0.81		
The fitness center provides various motivational programs to help me perform well in my exercises	0.82		
Exercise Flow Experience		0.91	0.68
I feel that time passes quickly while exercising at the fitness center	0.80		
I can concentrate well while exercising at the fitness center	0.85		
I do not have distracting thoughts while exercising at the fitness center	0.80		
I feel a sense of harmony between my thoughts and actions while exercising at the fitness center	0.84		
I feel joy while exercising at the fitness center	0.83		
Perceived Price Fairness		0.92	0.76
The usage price of the fitness center is reasonable	0.85		
The fitness center offers benefits that correspond to its usage price	0.89		
The usage price of the fitness center is appropriate	0.88		
The usage price of the fitness center is fair	0.88		
Rapport Formation		0.89	0.68
I have conversations with the staff to build a good relationship	0.75		
I generally have good communication with the staff	0.88		
I have a good relationship with the staff	0.87		
I remember the names of staff members	0.80		
Consumer Satisfaction		0.90	0.76
I am generally satisfied with the fitness center	0.88		
I am satisfied with the services provided by the fitness center	0.89		
The fitness center exceeds my expectations	0.83		
Consumer Loyalty		0.92	0.76
This would be my first choice when selecting a fitness center	0.86		
I will continue using this fitness center even if the prices increase	0.82		
I would recommend this fitness center if someone asks for advice	0.90		
I will tell my friends or colleagues about the positive aspects of this fitness center	0.89		
Exercise Involvement		0.84	0.64
I am highly interested in exercise	0.80		
Exercise is important to me	0.74		
Exercise is highly relevant to me	0.87		

Note: CR = composite reliability; AVE = average variance extracted.

**Table 3:** Correlations between constructs and AVE values.

	1	2	3	4	5	6	7	8	9
1 CS	**0.603**								
2 PS	*0.726*	**0.708**							
3 SS	*0.859*	*0.725*	**0.847**						
4 PF	*0.618*	*0.570*	*0.566*	**0.776**					
5 EF	*0.730*	*0.644*	*0.628*	*0.597*	**0.679**				
6 RF	*0.681*	*0.445*	*0.657*	*0.451*	*0.628*	**0.684**			
7 SAT	*0.817*	*0.722*	*0.729*	*0.841*	*0.741*	*0.545*	**0.755**		
8 LYT	*0.735*	*0.581*	*0.656*	*0.755*	*0.588*	*0.577*	*0.848*	**0.755**	
9 INV	*0.395*	*0.334*	*0.320*	*0.365*	*0.589*	*0.354*	*0.382*	*0.327*	**0.644**

Note: bold = AVE values; italics = correlations between the constructs; CS = core service; PS = physical servicescape; SS = social servicescape; PF = price fairness; EF = exercise flow; RF = rapport formation; SAT = satisfaction; LYT = loyalty; INV = exercise involvement.

**Table 4:** Results of hypothesis testing.

Path	Estimates	Standard Error	*p* Value	Results
CS → PF	0.669	0.031	<0.001	Accept
PS → EF	0.706	0.031	<0.001	Accept
SS → RF	0.682	0.032	<0.001	Accept
PF → SAT	0.642	0.034	<0.001	Accept
EF → SAT	0.353	0.039	<0.001	Accept
RF → SAT	0.150	0.034	<0.001	Accept
PF × INV → SAT	0.153	0.036	<0.001	Accept
EF × INV → SAT	−0.117	0.040	<0.01	Accept
RF × INV → SAT	0.061	0.034	=0.076	Reject
SAT → LYT	0.851	0.017	<0.001	Accept

Note: CS = core service; PS = physical servicescape; SS = social servicescape; PF = price fairness; EF = exercise flow; RF = rapport formation; SAT = satisfaction; LYT = loyalty; INV = exercise involvement.

## Data Availability

The data used for the current study are available from the corresponding author upon reasonable request.
